# The Pleiotropic Phenotypes Caused by an *hfq* Null Mutation in *Vibrio harveyi*

**DOI:** 10.3390/microorganisms11112741

**Published:** 2023-11-09

**Authors:** Yiqin Deng, Shujun Zang, Ziyang Lin, Liwen Xu, Changhong Cheng, Juan Feng

**Affiliations:** Key Laboratory of South China Sea Fishery Resources Exploitation & Utilization, Ministry of Agriculture and Rural Affairs, South China Sea Fisheries Research Institute, Chinese Academy of Fishery Sciences, Guangzhou 510300, China; yiqindd@126.com (Y.D.); zangshujun9969@163.com (S.Z.); Linziyang97@163.com (Z.L.); lwxu@163.com (L.X.); chengchanghong09@163.com (C.C.)

**Keywords:** *Vibrio harveyi*, Hfq, virulence, transcriptomics, small regulatory RNAs (sRNAs)

## Abstract

Hfq is a global regulator and can be involved in multiple cellular processes by assisting small regulatory RNAs (sRNAs) to target mRNAs. To gain insight into the virulence regulation of Hfq in *Vibrio harveyi*, the *hfq* null mutant, ∆*hfq*, was constructed in *V. harveyi* strain 345. Compared with the wild-type strain, the mortality of pearl gentian sharply declined from 80% to 0% in ∆*hfq* when infected with a dose that was 7.5-fold the median lethal dose (LD50). Additionally, ∆*hfq* led to impairments of bacterial growth, motility, and biofilm formation and resistance to reactive oxygen species, chloramphenicol, and florfenicol. A transcriptome analysis indicated that the expression of 16.39% genes on *V. harveyi* 345 were significantly changed after the deletion of *hfq*. Without Hfq, the virulence-related pathways, including flagellar assembly and bacterial chemotaxis, were repressed. Moreover, eleven sRNAs, including sRNA0405, sRNA0078, sRNA0419, sRNA0145, and sRNA0097, which, respectively, are involved in chloramphenicol/florfenicol resistance, outer membrane protein synthesis, electron transport, amino acid metabolism, and biofilm formation, were significantly down-regulated. In general, Hfq contributes to the virulence of *V. harveyi* 345 probably via positively regulating bacterial motility and biofilm formation. It is involved in flagellar assembly and bacterial chemotaxis by binding sRNAs and regulating the target mRNAs.

## 1. Introduction

*Vibrio harveyi* is a Gram-negative halophilic bacterium, which is common in coastal and estuarine environments. This bacterium is becoming one leading opportunistic pathogen that can cause fatal vibriosis in aquatic vertebrates and invertebrates [1]. Various marine fishes can be infected by *V. harveyi*, including European sea bass (*Dicentrarchus labrax*), grouper (*Epinephelus coioides*), Japanese flounder (*Paralichthys olivaceus*), Atlantic salmon (*Salmo salar*), rockfish (*Sebastes schlegeli*), and seabream (*Sparus aurata*) [2,3]. In particular, it is the main pathogen that causes the severe infection and death of grouper (*Epinephelus* sp.), which is the third largest cultured fishery species in China after yellow croaker and bass, with a yield of 192,045 tons in 2020 [4], and it is recognized to have a major constraint on production [5]. Therefore, an in-depth examination of its etiology is crucial to develop a strategy for the prevention and control of *V. harveyi* disease, especially to establish a strategy focused on blocking the virulence process, which has a great impact on the development of sustainable aquaculture.

Hfq, the global post-transcriptional regulator, has been reported to directly or indirectly regulate at least 20% of the genes in *Salmonella* spp. [6], 15% of the genes of *Pseudomonas aeruginosa* [7], and 6% of the genes of *Francisella tularensis* [8], *V. cholerae* [9], and *Yersinia pestis* [10]. It mediates the interactions of small regulatory RNAs (sRNAs) and target mRNAs, and thus plays important roles in the post-transcriptional regulation of many bacterial cell processes, including the production of virulence factors [11]. The virulence of *hfq* mutant strains is substantially attenuated in pathogens such as *Brucella abortus*, *Salmonella* spp., and *V. cholerae* [12]. The mutant usually shows clear reductions in growth, motility, stress and antibiotic resistance, biofilm formation, toxin production, or lipopolysaccharide (LPS) biosynthesis. Consequently, the *hfq* mutant is unable to invade and survive inside the host cell [12]. In *V. harveyi*, the interactions of Hfq and the quorum regulatory RNA Qrrs (Hfq-Qrrs) mediate the mRNA destabilization of the quorum-sensing master regulator, LuxR, and thus regulate biofilm formation [13]. However, the other regulatory functions of Hfq in *V. harveyi* are less known, especially its function in the regulation of virulence.

In the current study, the *hfq* null mutant and complemented strains were constructed in the virulent *V. harveyi* strain 345 [14]. In addition, we characterized the roles of Hfq in bacterial growth; motility; extracellular protease (ECP) activity; biofilm formation; resistance to HCl, H_2_O_2_, 2,2′-Bipyridine, and antibiotics; and fish infection in *V. harveyi*. Subsequently, a comparative transcriptome was carried out to explore the genes and pathways that were regulated by Hfq and likely contributed to the regulation of phenotype. The present findings may clarify the phenotypic and genotypic regulations of Hfq on the pathogensis in *V. harveyi*. The results will enrich the research of the pathogenic mechanism of *V. harveyi* and provide a theoretical basis for the prevention and treatment of vibriosis caused by *V. harveyi* infection.

## 2. Materials and Methods

### 2.1. Bacterial Strains and Media

All the strains used in this study are listed in Table 1. The *E.coli* strains were cultured in LB medium (containing 1% tryptone, 0.5% yeast extract, 1% sodium chloride, and 1.5% agar in solid medium). The tryptone, yeast extract, and agar were purchased from Thermo Fisher, Waltham, MA, USA, and the sodium chloride was purchased from Sango, Shanghai, China with 20 μg/mL chloramphenicol (Cm) when needed. The *V. harveyi* strains were cultured in LBS medium (salty LB medium; 2% sodium chloride is added to LB medium with a final sodium chloride concentration of 3%) with 34 μg/mL Cm when needed.

### 2.2. Gene Disruption and Complementation

The plasmida and the primers are, respectively, listed in Table 1 and Table 2. The wild-type *V. harveyi* was used to create the null mutant, Δ*hfq*, by employing allelic exchange, as previously described in [19]. Briefly, two homologous fragments (=1000 kb) at the up- and down streams of *hfq* were amplified using the *hfq*-UP-F/R and *hfq*-DOWN-F/R primers (Figure S1A), respectively. A suicide plasmid, pSW7848, was linearized by the pSW7848-F/R primers that contain small overlapping fragments from the two homologous fragments. Then, the two homologous fragments and the linearized pSW7848 were joined together via the ClonExpress Multis One Step Cloning Kit (Vozyme, Nanjing, China), and the recombinant pSW7848_∆*hfq* plasmid was finally generated. To carry out allelic exchange, an *E. coli* GEB883 donor strain carrying pSW7848_∆*hfq* was used to conjugate with the recipient strain, *V. harveyi* 345. The deletion of *hfq* was then validated via PCR (Figure S1B) and sequencing.

To complement the *hfq* mutation, the expression vector, pMMB207 [18], was linearized by the primer pair of pMMB207-F/R, and the complete *hfq*, including the ORF, the promoter, and the terminator, was amplified by the primer pair of com-*hfq*-F/R (Figure S1A). Then, the complete *hfq* was assembled into the linearized pMMB207 with the ClonExpress Multis One Step Cloning Kit (Vozyme, Nanjing, China), generating the recombinant plasmid, pMMB207_*hfq*. Subsequently, the recombinant plasmid was, respectively, transferred into the *hfq* deletion mutant and the wild-type *V. harveyi* 345 via conjugation [16], resulting in 345∆*hfq*:pMMB207_*hfq* and 345:pMMB207_*hfq*. The empty pMMB207 plasmid was transformed into an *hfq* deletion mutant, and the wild-type *V.harveyi* 345 was used as the control. The presence of an intact *hfq* gene was confirmed via PCR and sequencing.

### 2.3. Bacterial Growth

Single clones of WT (345:pMMB207), ∆*hfq* (345∆*hfq*:pMMB207), and C*hfq* (345∆*hfq*:pMMB207_*hfq*) were cultured overnight in LBS broth without antibiotics at 28 °C and 200 rpm. Then, the overnight culture was diluted 500-fold with 35 mL of fresh LBS medium and incubated with a 150 mL conical flask at 28 °C and 200 rpm. The OD600nm was measured every 1.0–3.0 h using a spectrophotometer (INESA, Shanghai, China).

### 2.4. Swimming Ability

Overnight cultures were prepared as mentioned above in “Bacterial growth”. Then, all the cultures were adjusted to OD600nm = 5.0. To test the swimming, 2.0 μL of each culture was spotted onto LBS agar plates containing 0.3% agar. The plates were incubated at 28 °C for 24 h, and the swimming diameters were measured.

### 2.5. Extracellular Protease (ECP) Activity Assay

Overnight cultures were prepared as in “Bacterial growth” and adjusted to OD600nm = 5.0 with fresh LBS broth. An amount of 2.0 μL of each dilution was spotted onto LBS agar plates containing 1.2% skimmed milk. The plates were incubated at 28 °C for 24 h. The extracellular protease activity was determined by measuring the diameter of the clear zone/diameter of the colony.

### 2.6. Measurement of Biofilm Formation

Overnight cultures were prepared as in “Bacterial growth” and adjusted to OD600nm = 1.0. Then, the adjusted cultures were diluted 100-fold with fresh LBS broth. An amount of 1 mL of the dilution was added in a well of a 48-well plate with three technical replicates of each strain, and fresh LBS medium was used as a control. The plate was incubated at 28 °C without shaking. The first stages of biofilm development (irreversible adherence and microcolony formation) was measured after incubation for 24 h as follows: The medium was sucked out; then, the attached cells were washed with 1 × phosphate-buffered saline (PBS) (Sango, Shanghai, China) and fixed with 10% methanol for 20 min. Subsequently, the biofilm was stained by 0.1% (*w*/*v*) crystal violet, rinsed with 1 × PBS, and eluted with 33% (*v*/*v*) acetic acid. Finally, the eluted dye was measured using a spectrophotometer at 570 nm.

### 2.7. Stress Response Assays

Overnight cultures were prepared as in “Bacterial growth” and diluted 300-fold with fresh LBS medium. In order to test bacterial resistance to acid, iron-restricted stress, and oxidative stress, the dilutions were cultured to OD600nm = 0.6–0.8, and then they were stressed with 9.0 mM HCl, 0.003% H_2_O_2_, or 1 mM 2,2′-Bipyridine (an iron-chelating agent) for 0.0 h, 0.5 h, and 1.0 h. The survival cell density (cfu/mL) was obtained by diluting and spreading on the LBS plate. The survival rates after being stressed for 0.5 h and 1.0 h were then calculated and normalized to the bacterial numbers prior to stress.

### 2.8. Antibiotic Resistance

A single colony of wild-type strain 345 and *hfq* mutant strain 345∆*hfq* were cultured overnight in LBS broth at 28 °C 200 rpm. Disk diffusion assays were carried out to measure bacterial antibiotic resistance. The overnight cultures were adjusted to OD600nm = 1.0, and 200 μL adjusted cultures were spread onto LBS agar plates. Antibiotic discs, including amoxicillin (20 μg/disk), ciprofloxacin (50 μg/disk), chloramphenicol (30 μg/disk), doxycycline (300 μg/disk), enrofloxacin (10 μg/disk), erythromycin (150 μg/disk), florfenicol (30 μg/disk), furazolidone (300 μg/disk), gentamicin (10 μg/disk), midecamycin (30 μg/disk), norfloxacin 10 μg/disk, rifampicin (5 μg/disk), tetracycline (30 μg/disk), tobramycin (10 μg/disk), trimethoprim-sulfamethoxazole (23.75/1.25 μg/disk), and vancomycin (30 μg/disk) (Hangzhou Binge Microorganism Reagent Co., Hangzhou, China), were placed on dried plates with sterilized tweezers. The plates were incubated at 28 °C for 24 h before measuring the inhibition zone, and the results were determined by referencing the Clinical and Laboratory Standards Institute (CLSI) [20].

### 2.9. Fish Infection Assay

All experimental procedures in the present study were conducted in accordance with the relevant guidelines and regulations of Committee on Laboratory Animal Welfare and Ethics of South China Sea Fisheries Research Institute (nhdf2023-08).

The LD50 of the WT to pearl gentian was determined via intraperitoneal injection. The pearl gentians with a weight of 50 ± 2 g/fish that were used for infection assay were purchased from a local aquaculture farm in Shenzhen, China. Before injection, the pearl gentians were stocked in aerated circulating seawater at room temperature for a two-week acclimatization. Pearl gentians were fed twice daily at 8:00–9:00 a.m. and at 3:00–4:00 p.m., and they were fasted for 3 days before injection. The WT strain was scribed on LBS agar plate with 34 µg/mL Cm and stationary incubated at 28 °C overnight. Then, a single clone of the WT strain was selected from the overnight plate and scribed on LBS glass slope with 34 µg/mL Cm, and then stationary incubated at 28 °C overnight. Then, the bacterial cells were re-suspended with 3 mL normal saline and adjusted to 8.45 × 10^8^, 8.45 × 10^7^, 8.45 × 10^6^, 8.45 × 10^5^, and 8.45 × 10^4^ cfu/mL, respectively. A total of 180 fishes were randomly divided into 18 equal parts and separately cultured in 500 L plastic buckets with 10 fish per bucket. Every 3 equal parts were divided as a group. There were totally 6 groups (five above-mentioned concentration gradient groups and one normal saline group). Intraperitoneal injection was carried out for those 6 groups with 100 μL/fish. The buckets were oxygenated, and the fish mortality was recorded for 7 days. The LD50 was calculated using the method of Sun Ruiyuan Käber with the formula of lgLD50 = XK − i(∑p − 0.5) (“XK” is the logarithm of the maximum dose, “i” is the logarithm of the ratio of two adjacent doses, and ∑p is the sum of mortality in each group).

With an intraperitoneal injection at a dose of 7.5-fold LD50, the effect of *hfq* deletion on the virulence of *V. harveyi* to pearl gentian was subsequently assessed. Individual clones of the WT, ∆*hfq*, and *Chfq* were cultured on LBS glass slopes with 34 µg/mL Cm, and then incubated at 28 °C overnight. The bacterial cells were then re-suspended with 3 mL normal saline. The cells were diluted into OD600nm = 1.85 (1.68 × 10^8^ cfu/mL) with normal saline. Each strain was injected with 3 buckets of fish (10 fish/bucket, and 100 μL cell or normal saline per fish). The buckets were oxygenated, and the fish mortality was recorded for 7 days.

### 2.10. Comparative Transcriptome Analysis

The mid-log bacterial cells (OD600nm = 3.5) of the wild-type strain *V. harveyi* 345 and the *hfq* mutant strain 345∆*hfq* were collected via centrifugation and immediately frozen in liquid nitrogen. Three replicates from each strain were collected and combined into a single operation for RNA-seq. The total RNA was then extracted using the TRIzol^®^ Reagent (Invitrogen, Carlsbad, CA, USA) in accordance with the manufacturer’s instructions, and DNase I (TaKara, Tokyo, Japan) was used to remove the genomic DNA. Next, the RNA quality was assessed using an Agilent USA 2100 Bioanalyzer and quantified using the ND-2000 (NanoDrop Technologies, Wilmington, NC, USA). Only high-quality RNA samples (OD260/280 = 1.8~2.0, OD260/230 ≥ 2.0, RIN ≥ 6.5, 28S:18S ≥ 1.0, ≥100 ng/μL, ≥2 μg) were employed to construct sequencing library.

The RNA-seq transcriptome library was created using 2 μg of total RNA and the TruSeq^TM^ RNA sample preparation kit (Illumina, San Diego, CA, USA). The paired-end RNA-seq library was sequenced with the Illumina Novaseq 6000 (Illumina Inc., San Diego, CA, USA). The fastp tool [21] was used to remove low-quality sequences, reads containing adaptor sequences, and reads containing more than 5% of N bases (unknown bases). The high-quality reads in each sample were mapped to the reference *V. harveyi* (PRJNA418027) genome via Bowtie2 [22]. Transcript per million (TPM) mapped reads was calculated via RSEM [23] to assess the gene expression.

The differentially expressed genes (DEGs) were identified by using the DESeq packages [24,25,26]. The difference in gene expression is considered to be significantly differentially expressed when the *p*-adjusted value (Padjust) of multiple tests is less than 0.05.

Compared with the whole genome background, an enrichment analysis of the Kyoto Encyclopedia of Genes and Genomes (KEGG) can determine the most important biological metabolic pathways and signal transduction pathways that DEGs are involved in. The cluster Profiler software, KOBAS 2.0, was used to perform a KEGG pathway enrichment analysis of DGEs [27]. BH-corrected *p*-value of ≤ 0.05 was the threshold for significant enrichment. The result showed the enrichment pathways with a corrected *p*-value of ≤ 0.5.

The software Rockhopper (version2.0.3) was used to predict sRNAs based on base sequencing coverage [28]. Then, the sRNAs were annotated via Blast, sRNAMap, sRNATarBase, SIPHT, and Rfam [29,30]. RNAfold was performed to predict sRNA secondary structure [31]. RNAphybrid and RNAplex were used to predict sRNA targets [32,33].

### 2.11. RT-qPCR Assay

All reagents were from Accurate Biolog Inc, Changsha, China. The total RNA of three replicates of each strain was extracted by using RNAiso Plus. DNase treatment and reverse transcription of 1 μg RNA of each replicate was conducted with the Evo M-MLV Mix Kit. RT-qPCR was then performed on a qTOWER3 84G (analytic jena) using the SYBR Green Pro Taq HS qPCR kit. The tested genes and specific primers are listed in Table S1. The 16s rRNA was used as an internal control, and the 2^−ΔΔCt^ method [34] was used to calculate the gene relative expression by normalizing to the value of the wild-type strain, *V. harveyi* 345.

### 2.12. Statistical Analysis

All phenotypic assays were carried out at least three times, and the results of one representative experiment are shown. One-way ANOVA was performed to examine variations in bacterial growth, motility, biofilm formation, stress response, and ECP activity. The *T* test was performed to examine the variations in antibiotic resistance and gene expression (significance level * *p* ≤ 0.05, ** *p* ≤ 0.01, *** *p* ≤ 0.001). The statistical analyses were conducted with IBM SPSS Statistics 19.0 [35].

## 3. Results

### 3.1. Growth Repression of ∆hfq

The *hfq* gene was successfully deleted from the chromosome of *V. harveyi* 345 to identify Hfq functions (Figure S1B). The complementation (Figure S1A) was constructed to confirm that the altered phenotypes could be restored via the re-introduction of *hfq*.

**Figure 1:**
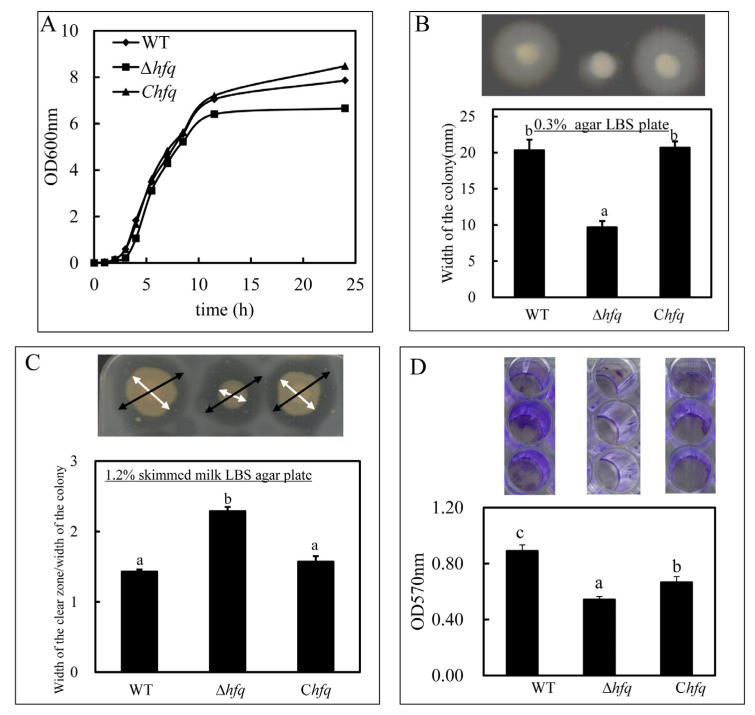
Bacterial growth (**A**), swimming motility (**B**), extracellular protease activity (**C**), and biofilm formation (**D**) of the wild-type strain (WT), the *hfq* mutant strain (∆*hfq*), and the complemented strain (C*hfq*). The values show the means and standard error of the mean (mean ± SEM, *n* = 3). Columns with different small letters, a, b and c, have significantly different one-way ANOVA results. The *p*-values of the one-way ANOVA of bacterial growth (**A**) were, respectively, 1.000, 0.003, 0.000, 0.005, 0.003, 0.030, 0.223, 0.174, 0.003, and 0.033 at 0, 1.0, 2.0, 3.0, 4.0, 5.5, 7.0, 8.5, 11.5, and 24 h. The *p*-values of the one-way ANOVA of swimming motility (**B**), extracellular protease activity (**C**), and biofilm formation (**D**) were, respectively, 0.001, 0.000, and 0.013. In (**C**), the black arrows are showing the width of the clear zone, and the white arrows are showing the width of the colony.

When the bacteria grew in the LBS medium, the lag phase of Δ*hfq* was nearly 1 h longer than that of the wild-type strain. The growth rate of the *hfq* mutant was similar to that of the wild-type strain in the exponential phase, and the total growth was lower than that of the wild-type strain (Figure 1A). The growth repression was restored in the complemented strain (Figure 1A).

### 3.2. Repressed Motility of ∆hfq

On a 0.3% agar LBS plate, ∆*hfq* showed a significantly decreased swimming motility (*p* = 0.001), and the motility was regained in the complemented strain (Figure 1B).

### 3.3. Increased Extracellular Protease Activity (ECP) of ∆hfq

The activities of the ECPs (diameter of the clear zone/diameter of the colony) of the wild-type strain (WT), the *hfq* mutant strain (∆*hfq*) and the complemented strain (C*hfq*) were 1.43 ± 0.03, 2.29 ± 0.06, and 1.57 ± 0.08, respectively (Figure 1C). The results indicated that the ECP activity was increased in the absence of the *hfq* mutant and significantly reduced upon *hfq* complementation (*p* = 0.000; Figure 1C).

### 3.4. Reduced Biofilm Formation of ∆hfq

As shown in Figure 1D, the biofilm was formed both on the bottom and walls of the 48-well plate after incubation for 24 h. In the *hfq* mutant, the biofilm was significantly decreased by the deletion of *hfq* (*p* = 0.013) and partly restored in C*hfq* (Figure 1D).

### 3.5. The hfq Mutant Increased Susceptibility to ROS

**Figure 2:**
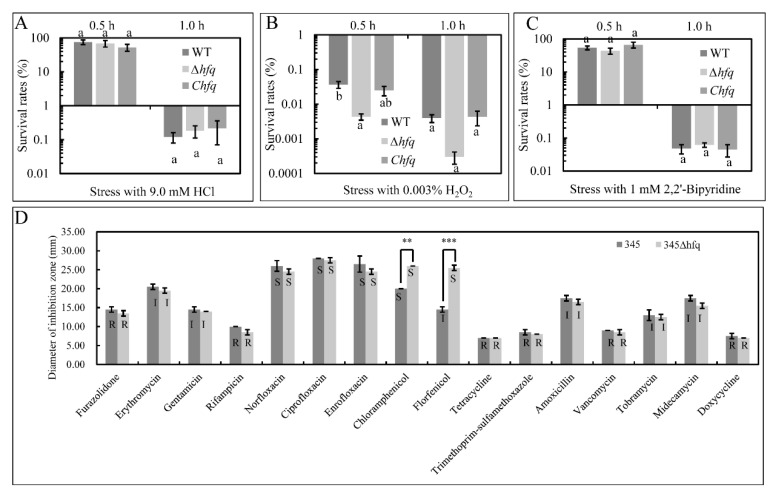
Bacterial stress response to 9.0 mM HCl (**A**), 0.003% H_2_O_2_ (**B**), and 1 mM 2,2′-Bipyridine (**C**) of wild-type strain (WT), the *hfq* mutant strain (∆*hfq*), and the complemented strain (C*hfq*) and the resistance to sixteen different antibiotics of the wild-type strain 345 and the *hfq* mutant strain, 345∆*hfq* (note: S, susceptible; I, intermediate; R, resistance to drug) (**D**). The values show the means and standard errors of the mean (mean ± SEM, n = 3). Columns with different small letters, a and b, have significantly different one-way ANOVA results. ** *p*-value ≤ 0.01; *** *p*-value ≤ 0.001.

As indicated in Figure 2A–C, compared to the WT, the mutant ∆*hfq* showed a similar survival after the exposure to 9.0 mM HCl or 1 mM 2,2′-Bipyridine, while demonstrating a greater sensitivity to the ROS of 0.003% H_2_O_2_ after 0.5 h of exposure. The survival rate was partly restored in C*hfq* (Figure 2B).

### 3.6. Loss of hfq Increases Sensitivity to Amphenicol Antibiotics (Chloramphenicol and Florfenicol)

Sixteen kinds of antibiotic discs were used to test the effect of *hfq* on antibiotic resistance. As shown in Figure 2D, both the wild-type strain and the *hfq* mutant showed similar levels of resistance or susceptibility to most antibiotics. However, the 345Δ*hfq* strain showed a significantly larger inhibition zone to the amphenicol class of antibiotics (chloramphenicol and florfenicol) than the wild-type strain.

### 3.7. Virulence Attenuation of ∆hfq

**Figure 3:**
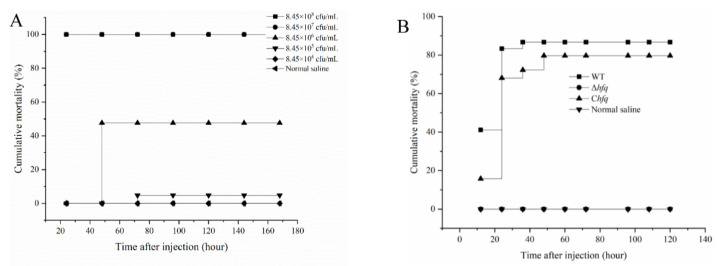
Fish mortality after being injected with *V. harveyi* wild-type strain (WT) with different concentrations (**A**) and fish mortality after being injected with *V. harveyi* wild-type strain (WT), *hfq* mutant strain (Δ*hfq*), and the completed strain (C*hfq*) with a dose of 7.5 LD50. In (**A**), the curves of 8.45 × 10⁷ cfu/mL and 8.45 × 10^8^ cfu/mL are overlapped. The curve of 8.45 × 10^6^ is overlapped with normal saline at 24 h and 48 h. The curve of 8.45 × 10^5^ is overlapped with normal saline at 24 h, 48 h, and 72 h. The curve of 8.45 × 10^4^ and normal saline are overlapped. In (**B**), the curves of Δ*hfq* and normal saline are overlapped.

When injected with 100 μL of different concentrations (8.45 × 10^8^, 8.45 × 10^7^, 8.45 × 10^6^, 8.45 × 10^5^, and 8.45 × 10^4^ cfu/mL) of WT bacterial cells, the total mortalities were 100%, 100%, 47.62%, 4.76%, and 0% in 7 days, respectively (Figure 3A). Based on the mortalities at different infectious doses, the LD50 was calculated as 4.49 × 10^4^ cfu/g for the WT strain. A fish infection assay was further conducted to evaluate the impact of *hfq* on the virulence of *V. harveyi* with an injection dose of 7.5 LD50 (Figure 3B). The fish that were injected with cultures of the WT and C*hfq* died rapidly within 12 h post injection (hpi), with mortalities of 41.11% and 15.74%, respectively, and within 24 hpi, they had mortalities of 83.33% and 68.06%, respectively. However, no fish died when they were injected with Δ*hfq* bacterial cells and normal saline. Finally, the groups of WT and C*hfq* reached mortalities of 86.7% and 79.6%, respectively, while the groups of Δ*hfq* and normal saline had 0% mortalities at 168 hpi.

### 3.8. Analysis of Differentially Expressed Genes

There was a total of 883 genes that were significantly differentially expressed in 345Δ*hfq* compared with the wild-type strain, *V. harveyi* 345, accounting for 16.39% of the total genes in *V. harveyi* 345. Among them, 538 were up-regulated, and 345 were down-regulated (Tables S2 and S3).

**Figure 4:**
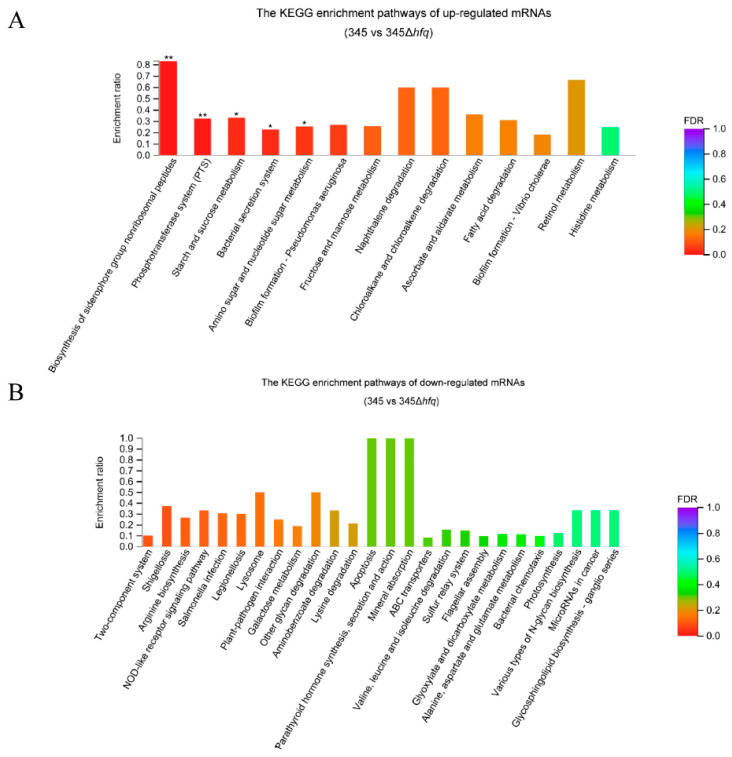
The KEGG enrichment pathways of up-regulated mRNAs (**A**) and down-regulated mRNAs (**B**). Corrected *p*-value. Corrected *p*-value of < 0.01 is **, and corrected *p*-value of < 0.05 is *.

A total of 14 pathways were up-regulated and enriched with a corrected *p*-value of ≤ 0.5, and 5 pathways were significantly enriched with a corrected *p*-value of ≤ 0.05 (*) or ≤0.01 (**) (Figure 4A). The up-regulated genes were enriched mainly in the pathways involved in metabolism, including biosynthesis of siderophore group nonribosomal peptides, phosphotransferase system (PTS), starch and sucrose metabolisms, amino sugar and nucleotide sugar metabolisms, fructose and mannose metabolisms, naphthalene degradation, chloroalkane and chloroalkene degradation, ascorbate and aldarate metabolisms, fatty acid degradation, retinol metabolism, and histidine metabolism. In addition, the pathways of the bacterial secretion system (especially the type III secretion system, including genes of *yscC*, *yscF*, *yscJ*, *yscL*, *yscT*, *yscU*, *yscV*, *yscP*, and type VI secretion system, including genes of *vasG*, *vasD*, *impK*, *impL*, *vgrG*, and *hcp*) and biofilm formation (especially the genes of *impM*, *impL*, *impC*, *impA*, *impB*, *impH*, *hcp*, *vasG*, *trpE*, *wcaJ*, *mshA*, *exoP*, *vpsT*, *vpsM*, *vpsQ*, *cdgC*, *vpsN*, *rp*oS, *hapR*, *ompU*, *ptsG*, and *ptsGb*) were also up-regulated with the deletion of *hfq* (Table S4).

No down-regulated pathways were significantly enriched, but 24 pathways were enriched with a corrected *p*-value of ≤ 0.5 (Figure 4B). The down-regulated genes were mainly involved in bacterial infection (*gapA* and *fliC*), apoptosis, flagellar assembly (*flgI*, *fliD*, *fliS*, *motY*, and *fliC*), bacterial chemotaxis (*malE*, *cheV*, *cheW*, and *mcp*), and microRNAs in cancer (*dcm*), which were related to bacterial virulence. The other down-regulated pathways were the signal transduction pathway of a two-component system and 15 different bacterial metabolism pathways.

**Figure 5:**
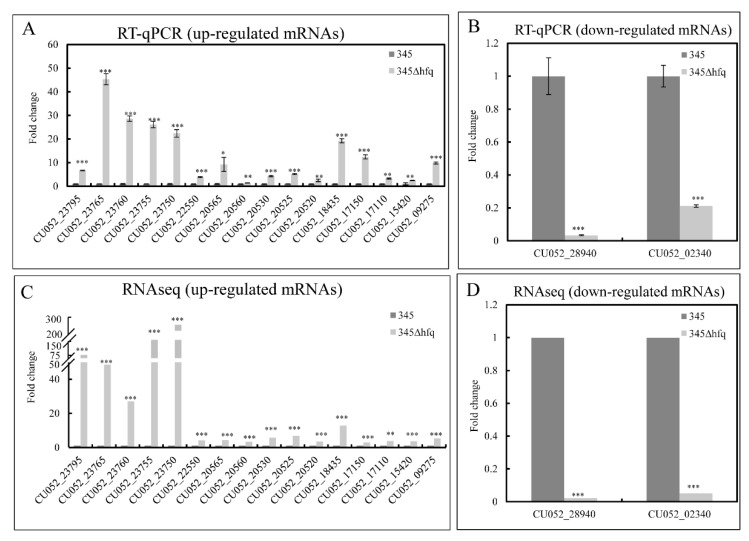
The gene expression ratio identified via RNAseq (**A**,**B**) and RT-qPCR (**C**,**D**). The gene expression was normalized to the value of the wild-type strain, *V. harveyi* 345. * *p* ≤ 0.05, ** *p* ≤ 0.01, *** *p* ≤ 0.001.

According to the results of the KEGG enrichment analysis, eighteen DEGs (16 up-regulated genes and 2 down-regulated genes) involving the bacterial secretion system, biofilm formation, apoptosis, and microRNAs in cancer were selected to confirm the correctness of the RNAseq data (Table S1). The results indicated that though the fold change was not exactly the same, the change trend was consistent between the results of the RNAseq and the RT-qPCR (Figure 5A–D and Tables S2 and S3). Therefore, the RNAseq results were convincing.

### 3.9. Prediction of Hfq-Dependent sRNAs

Hfq helps to stabilize the structures of sRNAs, and the sRNAs with reduced RNA levels in the absence of *hfq* may be Hfq-dependent sRNAs [36]. A total of 434 sRNAs were obtained in the transcriptome data (Table S5), and 11 sRNAs were significantly down-regulated (Padjust > 0.05, fold change > 8, and at least one of the strains had Transcripts Per Million reads (tpm) > 50) without *hfq* (Table S6). The sequences of sRNA0078, sRNA0145, and sRNA0097 were annotated as MicX, GcvB, and VqmR, respectively (Table S6). The CU052_28065 (*floR*) and CU052_11735 (*rsxE*) genes were, respectively predicated as the targets of sRNA0405 and sRNA0419 via both RNAphybrid and RNAplex (Table S6). In addition, the mRNA levels of the MicX target, *malG* [37]; the VqmR target, *vpsT* [38]; and the GcvB targets, *oppA* and *ndk* [39], were significantly up-regulated without *hfq* (Table S2).

## 4. Discussion

The pathogenic process of *Vibrio* includes adhesion, infection, colonization, reproduction, and toxin release. The pathogenic *Vibrio* will damage the host cells and tissues during invasion and growth and will interfere and destruct the normal metabolism or function of hosts with its metabolites (pathogenic factors) [40,41]. As an RNA chaperone, Hfq mainly plays its function at the post-transcriptional level by mediating the interaction between hundreds of regulatory non-coding sRNAs and their target mRNAs [36]. Hence, the loss of Hfq often results in pleiotropic phenotypes in many bacteria [12], with Vibrios not being exceptions. However, its role in *V. harveyi* is largely unknown. In the current study, the *hfq* mutant of *V. harveyi* was discovered to lose its virulence to pearl gentian. In vitro, phenotypes such as reduced motility [42], reduced biofilm formation [43], increased ECP activity [44], and increased sensitivities to ROS [45], chloramphenicol, and florfenicol [46] have been described to support the pathogenesis of Hfq in *V. harveyi*. In addition, the lost phenotypes were almost complemented by expressing Hfq with the expression vector, pMMB207, but not completely. As though the pMMB207 is low copy, it is more than a single copy of the chromosome [16]. Furthermore, the transcriptome was compared between the WT and Δhfq to investigate the gene level regulation associated with Hfq-affected phenotypes.

Growth and metabolism are the primary factors for bacteria to survive and cause infection. Corresponding to the weakened growth, in the current study, a large number of genes (enriched in 10 up-regulated pathways and 15 down-regulated pathways) and two sRNAs (sRNA0419 and GcvB) involved in metabolism were significantly differentially expressed. In addition, the GcvB target mRNAs, *oppA* (oligopeptide transport system substrate-binding protein) and *ndk* (nucleoside-diphosphate kinase) [36], were induced with the deletion of *hfq*. During bacterial pathogenesis, the first and most important step is to initially adhere to host cells. Then, the invasion after the adhesion enables the bacteria to escape the natural defense of the host, which enhances the pathogen’s colonization onto the host. Chen et al. [42] explain that flagella are important adhesion and motile organs, which play important roles in establishing the initial interaction with the host mucosal surfaces, including the skin, gills, and intestine, or cells. Additionally, flagella mediate bacterial chemotaxis and the response to the chemical concentration gradient of substances, making bacteria migrate towards environments that are favorable for growth and survival [47]. The motility mediated by the flagella of *Vibrio* spp. has been consistently related to their virulence. Berg and Singer [48] and Liang et al. [49] found that the decrease ij *V. cholera* motility not only reduced its colonization in the intestine but also reduced the expression of virulence factors such as cholera toxin and hemolysin. Therefore, in the current study, in *V. harveyi*, the attenuated swimming ability in *hfq* deletion could contribute to the reduced adhesion, invasion, and eventually, the virulence of Δ*hfq.* Furthermore, a comparative transcriptome indicated that the mRNAs enriched in flagellar assembly (*flgI*, *fliD*, *fliS*, *motY*, and *fliC*) and bacterial chemotaxis (*malE*, *cheV*, *cheW*, and *mcp*) are depressed, which should lead to the reduced swimming ability and, finally, reduced virulence in Δ*hfq*.

Following adhesion and colonization, biofilm formation allows for the pathogen to isolate from the outside and adapt to the host environment. Biofilm helps to reduce the production and even promote the enzymolysis of host cytokines, thus escaping from host immune defense. Additionally, biofilm can enhance bacterial tolerance to drugs, thus avoiding the disinfection and sterilization of drugs, including biotin [43]. In the current study, the decreased biofilm formation with the deletion of *hfq* in *V. harveyi* is similar to the results in *V. alginolyticus* and *E. coli*, but opposite to the condition in *Micrococcus catarrhalis* [12,50]. The genes involved in biofilm formation include *impM*, *impL*, *impC*, *impB*, *impA*, *impH*, *hcp*, *vasG*, *trpE*, *wcaJ*, *mshA*, *exoP*, *vpsT*, *vpsM*, *vpsQ*, *cdgC*, *vpsN*, *rp*oS, *hapR*, *ompU*, and *ptsG*; *ptsGb* (coming from the KEGG pathway: map02025 and map05111), was generally increased in the mid-log phase (OD = 3.5). Meanwhile, the sRNA *vqm*R that inhibits biofilm formation through the repression of *vpsT* was down-regulated. These results indicated the complex gene regulation of biofilm formation at different periods. For example, in *E. coli*, to both approach and move across the surface, flagellum-mediated swimming is required. Type I pili and the outer membrane protein, Ag43, are required for organism–surface interactions. Finally, colanic acid, a kind of extracellular polysaccharide (EPS), is needed for the development of normal *E. coli* biofilm architecture [51]. Therefore, the weakened motility probably contributes to the reduced biofilm formation in *V. harveyi* 345Δ*hfq.* More studies should be performed to analyze the phenotypes and gene expression during the same growth period to obtain the direct regulation mechanism at the genotype level.

The decreased resistance to oxidative stress with the deletion of *hfq* in *V. harveyi*, is similar to many studies of other Gram-negative bacteria, including *V. alginolyticus*, *B. abortus*, *E. coli*, *M. catarrhalis*, *Neisseria Meningitidi*, *Pseudomonas aeruginosa*, and *Yersinia pesti*s, while this is different from the results in the Gram-positive bacteria, including *Listeria monocytogenes* and *Staphylococcus aureus* [12]. As a kind of ROS, H_2_O_2_ can be produced by hosts during aerobic respiration, mediating bacterial killing. It kills microorganisms, probably via damaging bacterial DNA and membrane lipids. Thus, the resistance to ROS plays an important role in a pathogen’s adaptability to the host’s internal environment. These might explain the lower pathogenicity of the *hfq* mutant compared with the wild-type strain. In addition, the deletion of the *hfq* gene did not affect the acid (9 mM HCl) and iron (2,2-bipyridine) stress resistances of *V. harveyi*, which is different from those in *E. coli* and *Legionella pneumophila* [12]. Almost no loss of viability is seen after half an hour of treatment with HCl and 2,2-bipyridine, while most cells are dead after 1 h. We speculate that within half an hour, bacteria can still reach an acid–base balance by using proton pumps to literally pump protons out of the cell [52], and they can use the remaining iron to maintain growth [53]. After half an hour, the balance of proton transportation and iron usage are extremely disturbed, and the bacterial growth drops rapidly. However, the specific mechanism needs further study.

Antibiotics are widely used to prevent and kill pathogens by mixing them with feed [54]. Some hosts might be able to eliminate the invasive pathogens via their in situ symbiotic microorganisms that are capable of producing antibiotics as well [46]. Here, an sRNA, sRNA0405, was predicated to presumably target the gene, *floR*, a chloramphenicol and/or florfenicol efflux MFS, thus regulating chloramphenicol and florfenicol resistance. Though sRNA0405 was up-regulated, no significant changes were found in *floR*. Therefore, the regulation of sRNA0405 on *floR* may be growth-dependent, and it may not affect the expression of *floR* at the mid-log phase (OD600nm = 3.5), and a further study should be conducted to confirm the regulation of sRNA0405 on *floR.* Additionally, extracellular products secreted by *Vibrio* help to escape from the immune defense of the host before causing disease [55]. A further study by Wang et al. [44] demonstrated that the extracted extracellular product of *V. harveyi* is pathogenic to puffer fish. Among those extracellular secretions, proteases are the major substances that usually contribute to the virulence of *Vibrio* [56]. Lee [57] found that cysteine protease is a major exotoxin of pathogenic luminous *V. harveyi* in tiger prawn (*Penaeus monodon)*. However, two serine proteases from *V. metschnikovii* (Gamaleia) are exceptions that are not pathogenic to shrimp [58]. In our study, we have shown that the total level of proteases increased in the absence of *hfq* in *V. harveyi*, resembling that of proteases in *V. alginolyticus*, showing that the alkaline serine protease, Asp, and the total ECP were remarkably increased in the Δ*hfq* mutant, and its virulence to zebra fish was generally decreased [50]. Importantly, our study further demonstrated that the virulence of *V. harveyi* is completely lost when Hfq is absent. However, among those extracellular products, which secretion plays the primary role in the virulence of *V. harveyi* remains to be elucidated. In addition, the activities and physiological functions of specific extracellular proteases in *V. harveyi* require further investigation.

## 5. Conclusions

This study demonstrates the first piece of evidence that Hfq contributes to the virulence in *V. harveyi*, probably by maintaining bacterial growth, cellular mobility, biofilm formation, resistance to ROS, and some specific antibiotics, such as chloramphenicol and florfenicol. These observations are similar to several studies in different Gram-negative bacteria, while they are different from studies on Gram-positive bacteria, suggesting the different roles of Hfq between Gram-negative and Gram-positive bacteria [12,48,49,50]. Moreover, hundreds of genes have been found to be affected by the deletion of *hfq*. A KEGG analyses indicated that Hfq played variable roles in cell mobility, bacterial chemotaxis, biofilm formation, and protein secretion, all of which are associated with bacterial virulence through various pathway networks. In addition to these findings, more research is required to clearly elucidate the specific molecular mechanisms that deeply explain how Hfq affects bacterial virulence via different broadly regulated pathways, and most critically, how all the pathways work in concert.

Hfq has been extensively studied to act as an sRNA regulator that mediates post-transcriptional regulation, thus probably playing an essential role in bacterial virulence [15]. In this study, eleven sRNAs were shown to be significantly down-regulated in the *hfq* mutant, indicating that those sRNAs are probably Hfq-bonded and virulence-related sRNAs. The target prediction showed that they could affect bacterial metabolism, antibiotic resistance, acid resistance, outer membrane protein formation, biofilm formation, etc. Therefore, further studies remain to be investigated to understand the regulation of those sRNAs on the pathogenicity of *V. harveyi*. In addition, as Hfq is a key player in RNA-RNA transactions and post-transcriptional gene regulation, comparative proteomics should be conducted to reveal Hfq-dependent processes, because it integrates different RNA-based regulatory processes that may not be visible at the level of RNA abundance. Additionally, more research is required to discover new virulence-related genes, which are of great significance to control the infection of *V. harveyi*.

## Figures and Tables

**Table 1:** Strains and plasmids used in this study.

Strains or Plasmids	Relevant Characteristics	Sources
*V. harveyi*		
345	The wild-type strain *V. haveyi* 345	[14]
345∆*hfq*	*hfq* null mutant strain	This study
345:pMMB207 (WT)	Chloramphenicol resistance (Cm^r^); the wild-type strain *V. haveyi* 345 with the control plasmid pMMB207	This study
345∆*hfq*:pMMB207 (∆*hfq*)	Cm^r^; the *hfq* null mutant strain *V. haveyi* 345∆*hfq* with the control plasmid pMMB207	This study
345∆*hfq*:pMMB207_*hfq* (C*hfq*)	Cm^r^; *hfq* null mutant strain *V. haveyi* 345*hfq* with the complemented plasmid pMMB207_*hfq*	This study
*E. coli*		
Π3813	Emr^r^, Tc^r^, *lacIQ*, *thi1*, *supE44*, *endA1*, *recA1*, *hsdR17*, *gyrA462*, *zei298::tn10[Tc]*, Δ*thyA:: (erm-pir116)*; the intermediate host of suicide vector pSW7848	[15]
GEB883	Ery^r^, Tet^r^, WT *E.coli* K12 Δ*dapA::erm pir RP4-2* Δ*recA gyrA462*, *zei298::Tn10*; donor strain for conjugation	[16]
Plasmids		
pSW7848	Cm^r^; suicide vector with an R6K origin, requiring the Pir protein for its replication, and the *ccdB* toxin gene	[17]
pSW7848_∆*hfq*	Cm^r^; pSW848 containing the UP-DWON fragment of ∆*hfq*	This study
pMMB207	Cm^r^; expression vector	[18]
pMMB207_*hfq*	Cm^r^; pMMB207 containing intact *hfq* gene	This study

**Table 2:** Nucleotide sequences of primers used in this study.

Name	Sequence (5′-3′)	Purpose	Sources
pSW7848-F	GTCTGATTCGTTACCAATTATGACAAC	Linearization of pSW7848	[18]
pSW7848-R	GAATTCGATATCAAGCTTATCGATAC		
*hfq*-UP-F	aagcttgatatcgaattcCGGCGTTGATCTACAAAG	Amplification of hfq-UP	This study
*hfq*-UP-R	tcaataggaTTTATTTTCCTTATTTAATTTGTAGTTG		
*hfq*-DOWN-F	ggaaaataaaTCCTATTGAAGAACACTGTTAACC	Amplification of hfq-DOWN	This study
*hfq*-DOWN-R	ttggtaacgaatcagacGCATCAACAACATGTAACAAAATG		
Del-check-pSW7848-F	TCACTGTCCCTTATTCGCACC	Check the assembly of recombinant plasmid pSW7848_∆*hfq*	This study
Del-check-pSW7848-R	CTGCTTTTGAGCACTACCCG		
△*hfq*-check-F	CAGGCACTTGAAACCATGTCAG	Check the detection of *hfq*	This study
△*hfq*-check-R	CTCGCTCACCGGATTCATAAC		
pMMB207-F	AGAAGCGGTCTGATAAAACAGAATTTGC	Linearization of pMMB207	[18]
pMMB207-R	GCGCAACGCAATTAATGTAAGTTAG		
com-*hfq*-F	GCGATAACATTGAACAGGCAC	Amplification of hfq	This study
com-*hfq*-R	GCAATTTCTTGTGCCTTACCC		
com-PMMB207-check-F	CTACTGAGCGCTGCCGCACA	Check the complementation of *hfq*	This study
com-PMMB207-check-R	TCGTTTTATTTGATGCCTGGCAG		

## Data Availability

All sequence data that support the findings of this study were deposited in GenBank with the bioProject number of PRJNA801904 and the accession number of SRR178335621-SRR17833562.

## Supplementary Materials

The following supporting information can be downloaded at: https://www.mdpi.com/article/10.3390/microorganisms11112741/s1, Figure S1. Structure of the hfq locus (A) and the agarose gel electrophoresis results (amplified by the primers of △*hfq*-check-F/R) (B) of *Vibrio harveyi* 345 and the *hfq* mutant strain. The *hfq* gene is downstream of the *miaA* gene (locus ID = CU052_07575) and upstream of 3 genes (*hflX*, *hflK*, *hflC*) likely to form an operon. A putative Sigma70 promoter identified upstream of *hfq* is indicated by P. A total of 130 bp of hfq ORF (from the start codon ATG to 134 bp before the stop codon TAA of *hfq*) have been deleted, giving rise to the *V. harveyi* 345∆*hfq* strain. The stop condon was marked by triangle. This mutation does not affect the potential promoter of the operon, neither the ribosome binding site of the downstream gene. A wild type hfq gene was expressed by inserting the complete *hfq* (including the ORF, the promoter, and the terminator, which is amplified by the primer pair of *hfq*-F/R) into the expression vector pMMB207. Lane M is the DNA ladder, lane 2 is the PCR band of *V. harveyi* 345 strain amplified by the primers of △*hfq*-check-F/R, and lane 2 is the PCR band of *Vibrio harveyi* 345∆*hfq* strain amplified by the primers of △*hfq*-check-F/R. The other lanes are the results of other experiment; Table S1. The primers sequence for RT-PCR used in this study; Table S2. The up-regulated mRNAs in 345Δ*hfq* when campared with the wild type strain *V. harveyi* 345; Table S3. The down-regulated mRNAs in 345Δ*hfq* when campared with the wild type strain *V. harveyi* 345; Table S4. The genes of enriched KEGG pathways; Table S5. The predicated sRNAs in *V. harveyi* 345; Table S6. The down-regulated sRNAs in 345Δhfq when campared with the wild type strain *V. harveyi* 345 (least one strains’ tpm > 50 and the fc > 8).

## References

[1] Austin B., Zhang X.-H. (2006). Vibrio harveyi: A significant pathogen of marine vertebrates and invertebrates. Lett. Appl. Microbiol..

[2] Won K.M., Park S.I. (2008). Pathogenicity of Vibrio harveyi to cultured marine fishes in Korea. Aquaculture.

[3] Zhang X.-H., He X., Austin B. (2020). Vibrio harveyi: A serious pathogen of fish and invertebrates in mariculture. Mar. Life Sci. Technol..

[4] Liu X.Z., Cui L.F., Li S.M., Han X., Jiang K.Y., Yuan X.C., Yu X.J., Wang D., Wu F.X., Song D.D. (2021). China Fishery Statistical Yearbook.

[5] Shen G.M., Shi C.Y., Fan C., Jia D., Wang S.Q., Xie G.S., Li G.Y., Mo Z.L., Huang J. (2017). Isolation, identification and pathogenicity of *Vibrio harveyi*, the causal agent of skin ulcer disease in juvenile hybrid groupers *Epinephelus fuscoguttatus* × *Epinephelus lanceolatus*. J. Fish Dis..

[6] Ansong C., Yoon H., Porwollik S., Mottaz-Brewer H., Petritis B.O., Jaitly N., Adkins J.N., McClelland M., Heffron F., Smith R.D. (2009). Global Systems-level analysis of Hfq and SmpB deletion mutants in *Salmonella*: Implications for virulence and global protein translation. PLoS ONE.

[7] Sonnleitner E., Schuster M., Sorger-Domenigg T., Greenberg E.P., Bläsi U. (2006). Hfq-dependent alterations of the transcriptome profile and effects on quorum sensing in *Pseudomonas aeruginosa*. Mol. Microbiol..

[8] Meibom K.L., Forslund A.-L., Kuoppa K., Alkhuder K., Dubail I., Dupuis M., Forsberg A., Charbit A. (2009). Hfq, a novel pleiotropic regulator of virulence-associated genes in *Francisella tularensis*. Infect. Immun..

[9] Ding Y., Davis B.M., Waldor M.K. (2004). Hfq is essential for *Vibrio cholerae* virulence and downregulates σE expression. Mol. Microbiol..

[10] Geng J., Song Y., Yang L., Feng Y., Qiu Y., Li G., Guo J., Bi Y., Qu Y., Wang W. (2009). Involvement of the post-transcriptional regulator Hfq in *Yersinia pestis* virulence. PLoS ONE.

[11] Schu D.J., Zhang A., Gottesman S., Storz G. (2015). Alternative Hfq-sRNA interaction modes dictate alternative mRNA recognition. EMBO J..

[12] Chao Y., Vogel J. (2010). The role of Hfq in bacterial pathogens. Curr. Opin. Microbiol..

[13] Lenz D.H., Mok K.C., Lilley B.N., Kulkarni R.V., Wingreen N.S., Bassler B.L. (2004). The small RNA chaperone Hfq and multiple small RNAs control quorum sensing in *Vibrio harveyi* and *Vibrio cholerae*. Cell.

[14] Deng Y., Xu H., Su Y., Liu S., Xu L., Guo Z., Wu J., Cheng C., Feng J. (2019). Horizontal gene transfer contributes to virulence and antibiotic resistance of *Vibrio harveyi* 345 based on complete genome sequence analysis. BMC Genom..

[15] Le Roux F., Binesse J., Saulnier D., Mazel D. (2007). Construction of a *Vibrio splendidus* mutant lacking the metalloprotease gene vsm by use of a novel counterselectable suicide vector. Appl. Environ. Microbiol..

[16] Nguyen A.N., Disconzi E., Charrière G.M., Destoumieux-Garzón D., Bouloc P., Le Roux F., Jacq A. (2018). *csrB* gene duplication drives the evolution of redundant regulatory pathways controlling expression of the major toxic secreted metalloproteases in *Vibrio tasmaniensis* lgp32. Msphere.

[17] Val M.-E., Skovgaard O., Ducos-Galand M., Bland M.J., Mazel D. (2012). Genome engineering in *Vibrio cholerae*: A feasible approach to address biological issues. PLoS Genet..

[18] Liu J., Zhao Z., Deng Y., Shi Y., Liu Y., Wu C., Luo P., Hu C. (2017). Complete genome sequence of *Vibrio campbellii* LMB 29 isolated from red drum with four native megaplasmids. Front. Microbiol..

[19] Zhang Y., Deng Y., Feng J., Guo Z., Mao C., Chen H., Lin Z., Hu J., Su Y. (2021). CqsA inhibits the virulence of *Vibrio harveyi* to the pearl gentian grouper (♀ *Epinephelus fuscoguttatus*×♂ *Epinephelus lanceolatus*). Aquaculture.

[20] Wayne P.A. (2011). Performance Standards for Antimicrobial Susceptibility Testing.

[21] Chen S., Zhou Y., Chen Y., Gu J. (2018). fastp: An ultra-fast all-in-one FASTQ preprocessor. Bioinformatics.

[22] Langmead B., Salzberg S.L. (2012). Fast gapped-read alignment with Bowtie *Nat*. Methods.

[23] Li B., Dewey C.N. (2011). RSEM: Accurate transcript quantification from RNA-Seq data with or without a reference genome. BMC Bioinform..

[24] Anders S. (2010). Analysing rna-seq data with the deseq package. Mol. Biol..

[25] Love M.I., Huber W., Anders S. (2014). Differential analysis of count data–the deseq2 package. Genome Biol..

[26] Robinson M.D., McCarthy D.J., Smyth G.K. (2010). EdgeR: A Bioconductor package for differential expression analysis of digital gene expression data. Bioinformatics.

[27] Xie C., Mao X., Huang J., Ding Y., Wu J., Dong S., Kong L., Gao G., Li C.-Y., Wei L. (2011). KOBAS 2.0: A web server for annotation and identification of enriched pathways and diseases. Nucleic Acids Res..

[28] McClure R., Balasubramanian D., Sun Y., Bobrovskyy M., Sumby P., Genco C.A., Vanderpool C.K., Tjaden B. (2013). Computational analysis of bacterial RNA-Seq data. Nucleic Acids Res..

[29] Huang H.-Y., Chang H.-Y., Chou C.-H., Tseng C.-P., Ho S.-Y., Yang C.-D., Ju Y.-W., Huang H.-D. (2008). sRNAMap: Genomic maps for small non-coding RNAs, their regulators and their targets in microbial genomes. Nucleic Acids Res..

[30] Cao Y., Wu J., Liu Q., Zhao Y., Ying X., Cha L., Wang L., Li W. (2010). sRNATarBase: A comprehensive database of bacterial sRNA targets verified by experiments. RNA.

[31] Zhang M., Wong T.C. (1993). Solution conformation study of substance P methyl ester and [Nle10]-neurokinin A (4-10) by nmr spectroscopy. Biopolymers.

[32] Krüger J., Rehmsmeier M. (2006). RNAhybrid: microRNA target prediction easy, fast and flexible. Nucleic Acids Res..

[33] Tafer H., Hofacker I.L. (2008). RNAplex: A fast tool for RNA–RNA interaction search. Bioinformatics.

[34] Livak K.J., Schmittgen T.D. (2001). Analysis of relative gene expression data using real-time quantitative PCR and the 2^−ΔΔCT^ method. Methods.

[35] Kirkpatrick L.A. (2015). A Simple Guide to IBM SPSS Statistics-Version 23.

[36] Vogel J., Luisi B.F. (2011). Hfq and its constellation of RNA. Nat. Rev. Microbiol..

[37] Davis B.M., Waldor M.K. (2007). RNase E-dependent processing stabilizes MicX, a *Vibrio cholerae* sRNA. Mol. Microbiol..

[38] Papenfort K., Förstner K.U., Cong J.-P., Sharma C.M., Bassler B.L. (2015). Differential RNA-seq of *Vibrio cholerae* identifies the VqmR small RNA as a regulator of biofilm formation. Proc. Natl. Acad. Sci. USA.

[39] Sharma C.M., Papenfort K., Pernitzsch S.R., Mollenkopf H., Hinton J.C.D., Vogel J. (2011). Pervasive post-transcriptional control of genes involved in amino acid metabolism by the Hfq-dependent GcvB small RNA. Mol. Microbiol..

[40] Janda J.M. (1988). Current perspectives on the epidemiology and pathogenesis of clinically significant *Vibrio* spp.. Khirurgiia.

[41] Church S.R. (2015). Investigating Pathogenesis and Virulence of the Human Pathogen, Vibrio Vulnificus. http://hdl.handle.net/10871/17600.

[42] Chen Q., Yan Q., Wang K., Zhuang Z., Wang X. (2008). Portal of entry for pathogenic Vibrio alginolyticus into large yellow croaker Pseudosciaena crocea, and characteristics of bacterial adhesion to mucus. Dis. Aquat. Org..

[43] Rendueles O., Kaplan J.B., Ghigo J.M. (2012). Antibiofilm Polysaccharides. Environ. Microbiol..

[44] Wang B., Yu L.P., Yuan T., Jiang Z.Q. (2010). Pathogenicity of extracellular products of *Vibrio harveyi* to *Fugu obscurus*. J. Fish. Sci. China.

[45] Ransy C., Vaz C., Lombès A., Bouillaud F. (2020). Use of H_2_O_2_ to cause oxidative stress, the catalase issue. Int. J. Mol. Sci..

[46] Salminen S., Isolauri E. (2006). Intestinal colonization, microbiota, and probiotics. J. Pediatr..

[47] Bi S., Sourjik V. (2018). Stimulus sensing and signal processing in bacterial chemotaxis. Curr. Opin. Microbiol..

[48] Berg P., Singer M.F. (1995). The recombinant DNA controversy: Twenty years later. Proc. Natl. Acad. Sci. USA.

[49] Liang W., Pascual-Montano A., Silva A.J., Benitez J.A. (2007). The cyclic AMP receptor protein modulates quorum sensing, motility and multiple genes that affect intestinal colonization in *Vibrio cholerae*. Microbiology.

[50] Liu H., Wang Q., Liu Q., Cao X., Shi C., Zhang Y. (2011). Roles of *hfq* in the stress adaptation and virulence in fish pathogen *Vibrio alginolyticus* and its potential application as a target for live attenuated vaccine. Appl. Microbiol. Biotechnol..

[51] Markova J.A., Anganova E.V., Turskaya A.L., Bybin V.A., Savilov E.D. (2018). Regulation of *Escherichia coli* biofilm formation. Appl. Biochem. Microbiol..

[52] Bore E., Langsrud S., Langsrud Ø., Rode T.M., Holck A. (2007). Acid-shock responses in *Staphylococcus aureus* investigated by global gene expression analysis. Microbiology.

[53] Mey A.R., Wyckoff E.E., Kanukurthy V., Fisher C.R., Payne S.M. (2005). Iron and fur regulation in *Vibrio cholerae* and the role of fur in virulence. Infect. Immun..

[54] Jukes T.H. (1971). The present status and background of antibiotics in the feeding of domestic animals. Ann. N. Y. Acad. Sci..

[55] Defoirdt T. (2013). Virulence mechanisms of bacterial aquaculture pathogens and antivirulence therapy for aquaculture. Rev. Aquac..

[56] Osei-Adjei G., Huang X., Zhang Y. (2018). The extracellular proteases produced by *Vibrio parahaemolyticus*. World J. Microbiol. Biotechnol..

[57] Liu P., Lee K. (1999). Cysteine protease is a major exotoxin of pathogenic luminous *Vibrio harveyi* in the tiger prawn, *Penaeus monodon*. Lett. Appl. Microbiol..

[58] Kwon Y.T., Kim J.O., Moon S.Y., Yoo Y.D., Rho H.M. (1995). Cloning and characterization of the gene encoding an extracellular alkaline serine protease from *Vibrio metschnikovii* strain RH530. Gene.

