# Supplementary figures and images for: The Pleiotropic Phenotypes Caused by an hfq Null Mutation in Vibrio harveyi
Source: Microorganisms. 2023 Nov 9;11(11):2741. doi: 10.3390/microorganisms11112741 (PMC10672845; doi:10.3390/microorganisms11112741)

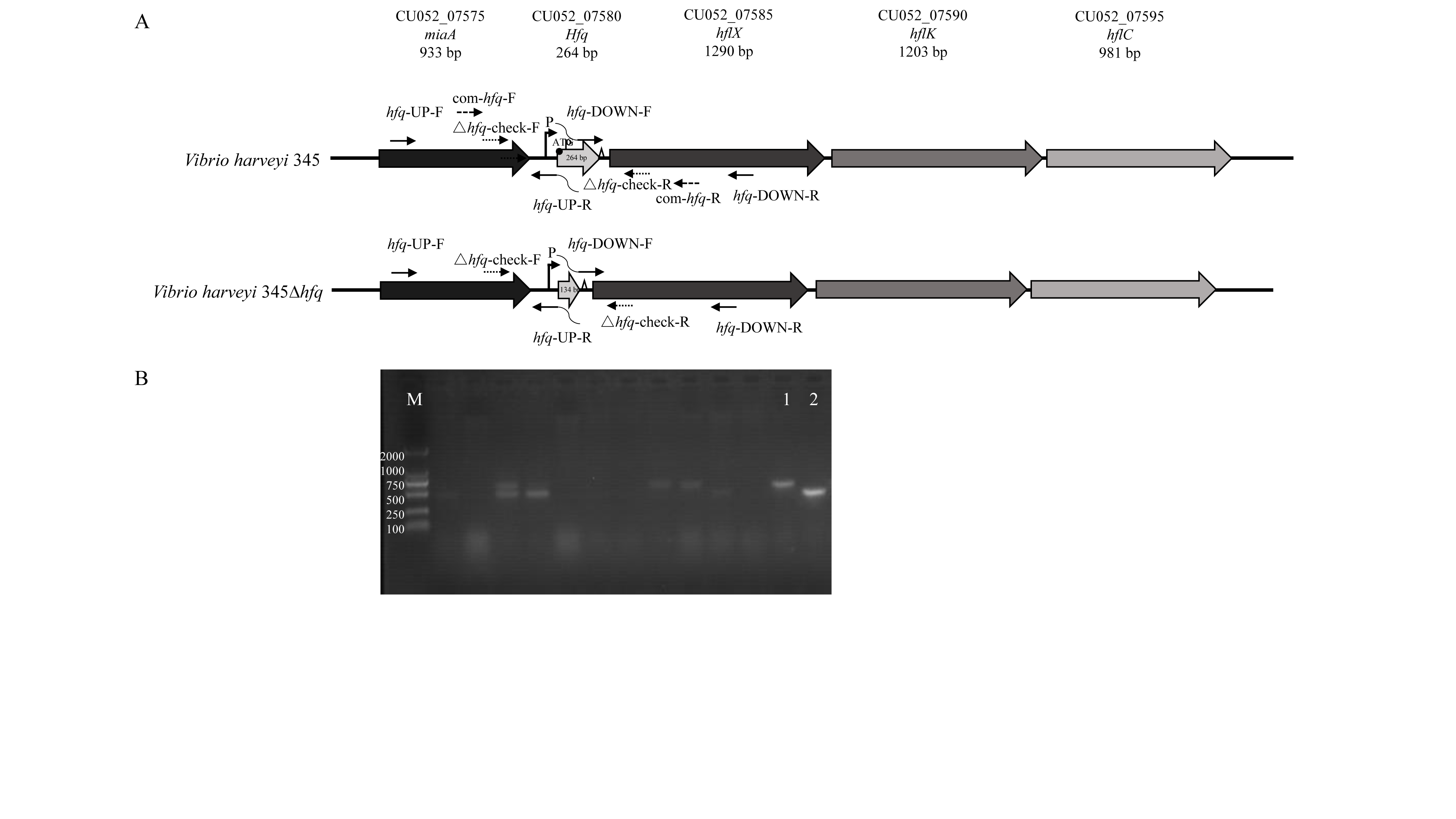

Supplement: Supplementary file 1 [file microorganisms-11-02741-s001.zip › Figure S1.tif]
